# Effects of Carbon/Kevlar Hybrid Ply and Intercalation Sequence on Mechanical Properties and Damage Resistance of Composite Laminates under Quasi-Static Indentation

**DOI:** 10.3390/polym16131801

**Published:** 2024-06-25

**Authors:** Mingling Wang, Zhongxiang Pan, Qimao Cai, Lei Zhao, Zhenyu Wu

**Affiliations:** 1 Faculty of Mechanical Engineering, Zhejiang Sci-Tech University, Hangzhou 310018, China; 2 College of Textile Science and Engineering, Zhejiang Sci-Tech University, Hangzhou 310018, China; 3 School of Architecture and Engineering, Zhejiang Sci-Tech University, Hangzhou 310018, China

**Keywords:** laminates, hybrid intercalation, damage resistance, Quasi-Static Indentation

## Abstract

The investigation of damage development is essential for the design and optimization of hybrid structures. This paper provides a reference for the structural design of brittle–ductile hybrid LVI-resistant laminates through analyzing the damage development mechanism of carbon/Kevlar fabric-reinforced composite laminates. The effects of Kevlar fabric hybrid ply and intercalation on the damage development of carbon/Kevlar composite laminates under low-velocity impact (LVI) were investigated using quasi-static indentation (QSI). It was found that an increase in the Kevlar hybrid ratio significantly reduced the peak load and stiffness of these laminates (the maximum decreases in strength and stiffness were 46.03% and 41.43%, respectively), while laminates with identical hybrid ratios but different plying configurations maintained a comparable stiffness under QSI, with differences of less than 5%. Interestingly, Kevlar fibers exhibited irregular fractures as the yarn was splitting, while carbon fibers presented neat breaks, which indicated material-specific failure modes. Notably, the introduction of Kevlar hybridization beyond pure Kevlar configurations (KKKK) resulted in a decrease in the percentage of fiber damage (CCCC, CCCK, CCKK, and KCCK accounted for 80%, 79.8%, 70%, and 60% of fiber damage, respectively), attributed to an increase in resin cracks and lower levels of Kevlar yarn breakage. The internal damage diameter of specimens was accurately predicted from the diameter of visible damage on the QSI surface. Compared with CCCC and CCKK setups, which are affected by resin cracks formed via the carbon surface on the loading side propagating along the yarn direction (including the yarn settling direction), KCCK demonstrated less delamination between the first and second ply.

## 1. Introduction

Carbon fiber-reinforced polymer (CFRP) composite laminates have the advantages of high strength, high modulus, and light weight, making CFRPs widely used in the aerospace and automotive industries [1]. However, CFRPs are sensitive to transverse low-velocity impact (LVI) load [1], which reduces their strength. A hybrid design has been proven to be an effective way to improve the mechanical properties of materials [2,3,4,5] and particular hybrid fiber designs including glass fiber, PP, natural linen fiber, etc., have been reported by numerous researchers [6,7] to improve the low-velocity impact resistance of carbon fiber laminates. Among these, Kevlar fiber is used in military uniforms due to its excellent toughness [8]; its use has grown in popularity in the last few years. The unidirectional [9,10], sandwich [11], three-dimensional orthogonal [12], plain weave [13], and 3D-printed unidirectional [14] forms of Kevlar/carbon hybrid structures have been extensively investigated under LVI, ballistic, and high-speed impact loading. For example, Gustin et al. [11] found that the distribution of Kevlar on the low-velocity impact side could enhance the energy absorption of sandwich laminates but reduced the stiffness of structures. Dorey et al. [9] demonstrated that Kevlar/carbon fiber hybrid materials exhibited a better overall impact performance compared with single-material laminate structures. Sojan Andrews Zachariah et al. [15] found through Charpy impact tests that laminates with two plies of aramid fabric at (0/90°) and (±45°) orientations on their outer surface displayed improved impact behavior in terms of average impact strength and absorbed energy. These findings indicate that different hybrid ply configurations affect the impact performance of specimens [16]. Therefore, the influence of hybrid ply number [17] and intercalation sequence [18] on the LVI performance of Kevlar/carbon fiber woven plain structures was investigated; it was found that the impact performance of carbon laminates could be improved through utilizing the appropriate Kevlar ply configuration. However, previous studies were limited by their short duration of LVI load, making it challenging to assess the damage evolution within each component of hybrid specimens through experimental methods during the loading process. Significantly, explicitly understanding the initiation and propagation processes of damage in hybrid composite materials and recognizing the failure modes of each component under loading [19,20] are essential for targeted structural optimization and design [21]. Therefore, it is necessary to investigate the influence of Kevlar plies and their configuration on the damage evolution of carbon fiber/Kevlar laminates.

Consequently, many researchers [22] have found that the damage caused by quasi-static indentation (QSI) is similar to the damage caused by LVI [23,24]. Therefore, they attempted to obtain more evidence of damage events during LVI via QSI testing [25,26]. Furthermore, compared with LVI, QSI testing can provide more precise real-time data of damage initiation and propagation [27]. For instance, Lammerant et al. [28] analyzed the interaction between matrix cracks and the delamination of composites under LVI loads via QSI tests. Using QSI, Cesari et al. [29] reported the damage progression in laminates of different stacking sequences under LVI loads. Sirichantra [30] determined that the flaps in thin woven fabric-reinforced CFRP panels could be predicted using QSI experiments. Thus, QSI is considered as an effective way to optimize the stacking sequences or designs of composite materials such as thin laminates or hybrid structures [26]. 

Observation techniques such as acoustic emission (AE) technology and X-ray micro-computed tomography (μCT) can be employed to observe the progressive development of damage [31]. Various damage mechanisms [32], including matrix cracking, delamination, and fiber fracture, particularly distinguishable via amplitude signals, can be identified through AE technology. Additionally, μCT is an advanced and non-destructive evaluation technique that has been applied in numerous studies [33,34], offering information in relation to the internal damage distribution of specimens. Above all, AE monitoring technology and μCT have been applied to obtain evidence of the damage modes and internal damage of specimens during QSI testing.

Therefore, the effect of Kevlar plies (the hybrid ratio and the order of Kevlar interlacing positions under the same hybrid ratio) on the development of the in-plane progressive damage of carbon fiber fabric laminates during out-of-plane QSI load, based on AE and μCT techniques, was investigated. The stiffness and strength of carbon/Kevlar plain fabric or triaxial fabric laminates were measured. The dominant damage modes in each stage of the progressive damage process were analyzed and the relationship between the maximum diameter of the internal damage volume and the apparent surface damage area was established. This study aimed to provide a reference for the structural design of brittle–ductile hybrid LVI-resistant laminates via analyzing the damage development mechanism of carbon/Kevlar fabric-reinforced composite laminates.

## 2. Experiment

### 2.1. Materials

#### 2.1.1. Composite Fabrication

**Figure 1:**
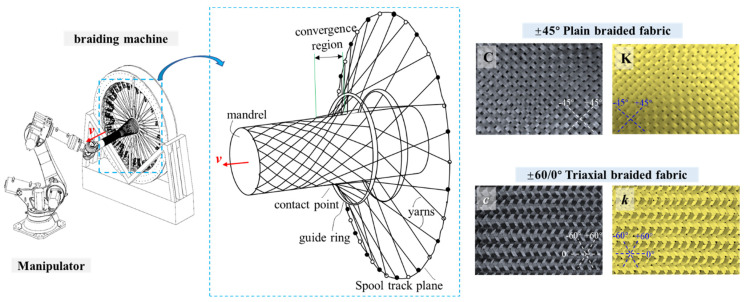
The automatic overbraiding approach to braid the plain and triaxial fabric reinforcements.

Considering its advantages of high flexibility in woven fabric design and minimal yarn wear during the knitting process, an automatic overbraiding machine was used to prepare carbon fiber fabrics (T700SC-12 K carbon fiber, Toray, Tokyo, Japan) and Kevlar fiber fabrics (Kevlar 49-5840D, Dupont, Wilmington, DE, USA). There were +45° and −45° braided yarns interlaced in the plain fabric, while there were +60°, −60°, and 0° braided yarns interlaced, respectively, in the triaxial fabric. A schematic diagram of the braiding equipment and fabrics is shown in Figure 1, while the parameters of the fibers are reported in Table 1. Carbon fiber demonstrates high strength and modulus, accompanied by limited elongation, indicating its high tensile strength and brittleness. In contrast, Kevlar exhibits exceptional elongation at the break, while retaining a certain level of strength, suggesting that Kevlar possesses a superior toughness compared with carbon fibers. Consequently, interlacing Kevlar and carbon fiber fabrics can enhance the mechanical properties of single-fiber structures. 

#### 2.1.2. Specimens

The composite laminates were manufactured using vacuum-assisted resin transfer molding (VARTM) with the epoxy resin EPOLAM 2040 and the hardener liquid system EPOLAM 2042 (AXSON Co., Ltd., Saint-Ouen L`Aumone, France). The epoxy resin and hardener liquid system were mixed and cured at room temperature for 24 h and then at 65° for 16 h. The laminate specimens were cut into 150 × 100 mm sections for QSI tests, for consistency with the LVI tests. To mitigate the effects of the preparation and cutting process, all specimens were cured and cut using the same equipment under identical curing conditions and within a consistent temperature and humidity range. The configurations of plain and triaxial fabric-reinforced composite specimens are reported in Table 2. 

### 2.2. Quasi-Static Indentation (QSI) Tests

**Figure 2:**
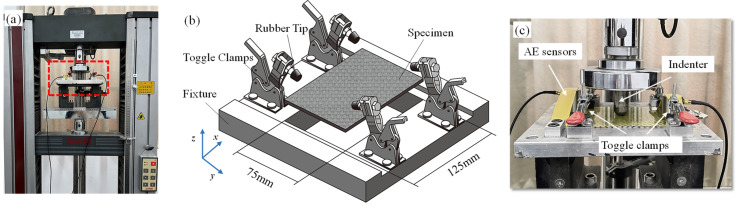
(**a**) Quasi-static indentation (QSI) device; (**b**) schematic diagram of fixture; (**c**) specimen and fixture of QSI test.

Considering the differences in damage modes caused by boundary conditions, the fixture and indenter of the QSI test were consistent with those of previous LVI studies. QSI tests were conducted using a servo–hydraulic testing machine with a *Φ*19 mm spherical head indenter at the center of the specimens, as shown in Figure 2a. The specimens were supported over a 125 × 75 mm^2^ rectangular hollow window fixture and were held with four toggle clamps at their four corners (Figure 2b). In accordance with Ref. [35], the tests were carried out using displacement control, at a constant rate of 2 mm/min. AE sensors were placed on the surface of the specimens (Figure 2c) to obtain their AE signals under the loading process; the AE signals were analyzed to distinguish damage mechanisms including matrix cracking, delamination, and fiber fracture. The QSI process was divided into four deflection levels (deflection is the displacement of the indenter during the test) to explore damage development under different deflections. Each specimen was tested at least three times in all tests and the permissible error of the results was controlled within 5%.

## 3. Results and Discussion

### 3.1. Load–Deflection Curve

**Figure 3:**
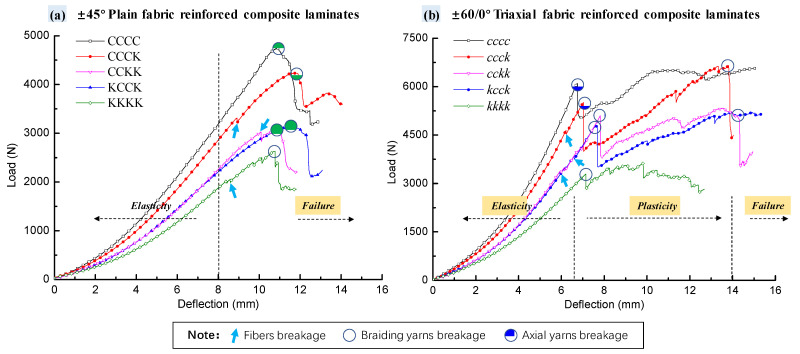
Load–deflection curves of composite laminates with different hybrid plies and intercalation orders of carbon/Kevlar laminates under QSI: (**a**) ±45° plain fabric-reinforced; (**b**) ±60/0° triaxial fabric-reinforced.

The load–deflection curves of carbon/Kevlar composite laminates with different Kevlar hybrid plies and intercalation under QSI tests are exhibited in Figure 3.

For plain woven fabric-reinforced laminates, as shown in Figure 3a, the load–deflection curves reflected the elastic response characteristics during the initial stage of loading (where deflection was less than 8 mm); then, the curves jittered (as marked with the blue arrows in Figure 3a) and continued to rise to the peak value during the loading process. 

It is generally believed that curve fluctuations are caused by resin and fiber breakage, while larger fluctuations are caused by damage to fiber bundles. However, the load–deflection curve stage and damage morphology stage are used in this paper, as shown in Figures 7c,e and 11 and Table 4. It was concluded that the jitter highlighted with blue arrows was the pull-out damage of the Kevlar fiber bundle on the reverse side. After the peak load, the laminates entered the failure stage of rapid degradation of the bearing capacity (as marked with the green circles in Figure 3a), corresponding to the yarn’s rupture (as shown in Figure 5). 

The load–deflection curves of the triaxial fabric-reinforced laminates are shown in Figure 3b. The curves presented elastic characteristics when the deflection was less than 6.5 mm and then, the curves jittered slightly (as marked with the blue arrows in Figure 3b), which was caused by resin cracking and fiber pull-out. The load continued to rise until the first peak (as marked with the purple circles in Figure 3b), even with minor fiber damage, after which the bearing capacity of the triaxial fabric-reinforced laminate suddenly dropped sharply as the backing yarn fractured. However, unlike the plain laminate, the bearing capacity of the triaxial laminate continued to rise slowly with the increase in deflection (entering the structural plastic stage) until the arrival of the second peak load (as marked with the green circles in Figure 3b). This is likely to have been due to the fact that the other two interlaced yarns of the triaxial laminate continued to bear the load after the rupture of the backing yarn, until the next interlocking yarn rupture occurred, resulting in a second degradation of the load. Additionally, the linear stage of the triaxial structure was shorter than that of the plain woven structure. Compared with the plain woven structure, the axial yarns effectively shared the punch stress and prevented the significant tensile deformation of the interlocking yarns on the reverse side. 

**Figure 4:**
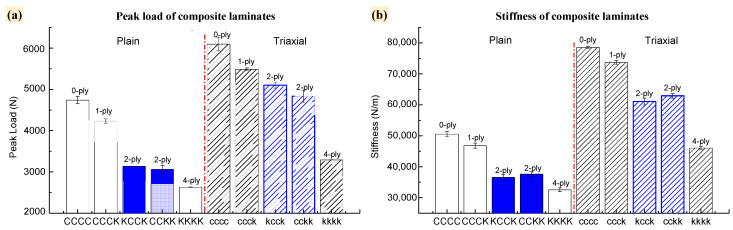
Composite laminates with different hybrid plies and intercalation order of carbon/Kevlar laminates under QSI: (**a**) peak load of specimens; (**b**) stiffness of specimens.

As shown in Figure 4, the pure carbon fiber configuration (whether plain woven fabric-reinforced laminate (CCCC) or triaxial fabric-reinforced laminate (*cccc*)) had better static mechanical advantages in terms of stiffness and strength compared with the carbon/Kevlar hybrid laminates; the peak load and stiffness of both the plain and triaxial fabric-reinforced laminates were significantly reduced with the increase in Kevlar hybrid ply. As displayed in Table 3, compared with CCCC, the peak load decreased by 10.72%, 35.42%, and 44.51%, respectively, while the stiffness decreased by 7.35%, 25.59%, and 35.55%, respectively, when the Kevlar ply number on the reverse side of the plain laminates was 1, 2, or 4. When the Kevlar ply distributed on the back side of the triaxial laminates was 1-ply, 2-ply, or 4-ply, the peak load decreased by 9.97%, 20.69%, or 46.03%, respectively, while the stiffness decreased 6.23%, 19.95%, or 41.43%, respectively, compared with cccc. As the proportion of Kevlar in the hybrid specimens increased, the lower modulus and strength characteristics of Kevlar played a dominant role, resulting in the decrease in strength and stiffness of the specimens. 

In addition, under the same Kevlar hybrid ply (carbon/Kevlar = 50%), the peak load and stiffness differences between KCCK and CCKK were 1.56% and 2.01%, respectively. The differences in peak load and stiffness between *kcck* and *cckk* were 4.38% and 2.22%, respectively. The difference in stiffness and strength between the two types of fabric-reinforced structures with the same Kevlar hybrid ply but different intercalation orders was less than 5%. Stiffness and strength in the elastic stage were related to the fiber type and fiber hybrid ratio; the fiber type and fiber hybrid ratio were similar when the specimens had same Kevlar hybrid ply, resulting in similarities in stiffness and strength.

### 3.2. Damage Morphology

**Figure 5:**
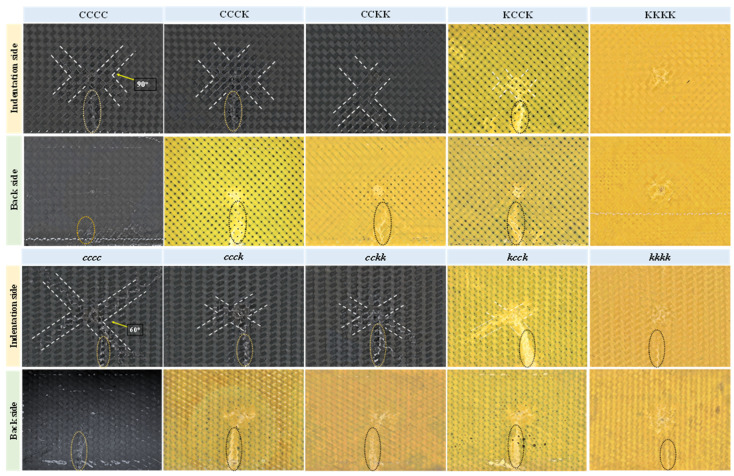
Apparent damage morphology of specimens on both the indentation side and the reverse side after the QSI test.

The apparent damage morphology of specimens on the indentation side and the reverse side is exhibited in Figure 5. It was found that the macroscopic damage on the indentation surface was distributed in an X-shaped crack along braided yarns, while there was no significant X-shaped crack propagation on the reverse side. The indenter acted on the center of the composite laminates in a vertical downward direction in the out-of-plane direction during the QSI process; the braided yarns on the diagonal of the rectangular specimens played a major bearing role under the four toggle clamps on the indentation side. Therefore, numerous resin cracks and considerable fiber damage along braided yarns appeared on the indentation surface, resulting in the appearance of X-shaped damage. As the interlaced angle between the triaxial and plain woven laminates differed, the X-shaped damage angle of the indentation surface of the triaxial laminates was 60 degrees or 120 degrees, while that of the plain woven laminate was approximately 90 degrees. Additionally, under the pressure of the punch, the back of the specimens was fixed to the rectangular support window, ultimately exhibiting damage characteristics similar to punching.

**Figure 6:**
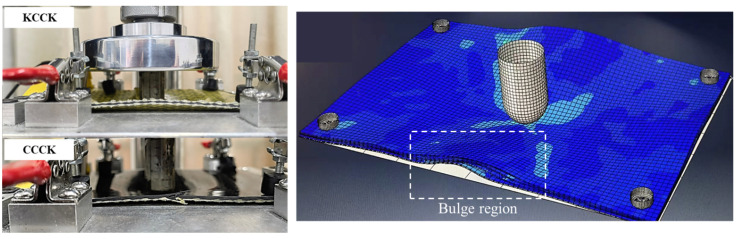
Characteristics of the bulge deformation of the composite laminate at the edge of the fixture during QSI load.

In addition, the clamps near the four corners of the rectangular specimens inhibited the vertical upward displacement trend of the specimen corners, which led to the bulge deformation of the specimen’s long side, as shown in Figure 6 (marked by a rectangle) and also caused bulge damage from the dented position of the indenter to the long sides of the rectangle specimens. However, due to the excellent deformation ability of Kevlar yarns, the bulge damage was not significant in the pure Kevlar structure.

### 3.3. Microscopic Damage of Plain Woven Structure

**Figure 7:**
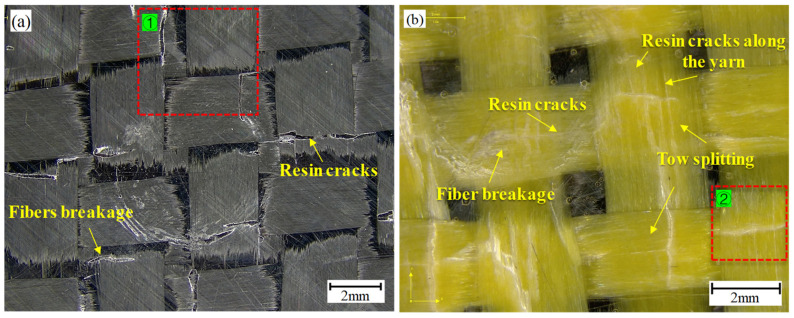

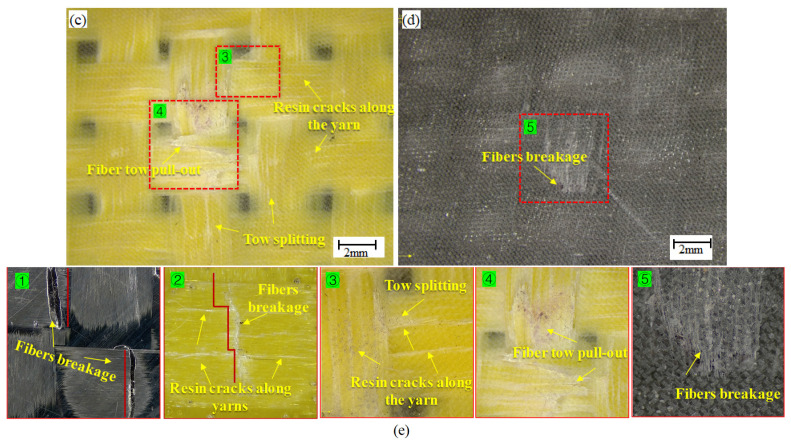
Microscopic damage analysis: (**a**) indentation side of CCCC; (**b**) indentation side of KCCK; (**c**) reverse side of CKKK; (**d**) reverse side of CCCC; (**e**) local amplification views.

When the carbon fiber surface was distributed on the indentation side, a brittle fracture of the carbon fiber bundle occurred, with a neat fracture and obvious resin crack damage, as shown in Figure 7a,e1. When Kevlar fibers were laid on the indentation side, the obvious resin crack was reduced and a micro-diffuse resin crack appeared inside the yarn. In addition, the Kevlar fiber bundle split, resulting in the irregular fracturing of the Kevlar yarn, as shown in Figure 7b,e2. The resin crack inside the yarns and the splitting of the yarn bundle can also be observed on the Kevlar surface, as shown in Figure 7c,e3,e4; the fracture of Kevlar yarn on the reverse was more irregular due to the tensile stress. When the carbon surface was on the reverse side, fiber breakage caused by the carbon fiber bundle pulling out was observed, as shown in Figure 7d,e5. It is noteworthy in the comparison of Figure 7c,d that when Kevlar was on the reverse side, Kevlar yarns broke at the interlaced position and adjacent interlaced points along the main load-bearing yarn. When the reverse side was the carbon fiber surface, it showed yarn breakage at only a single interlaced point. Possible reasons for these differences may have been that the brittle carbon fibers found it difficult to extend the stress to the adjacent interlaced points along the direction of the main load-bearing yarn and that fiber bundle fracture failure occurred.

**Figure 8:**
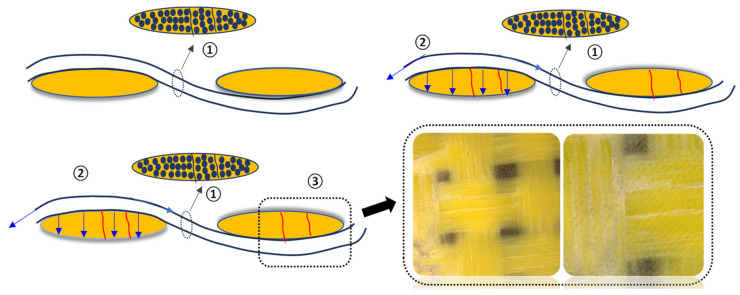
Damage development of Kevlar distributed on the reverse side. Key: ① Resin cracks inside the yarn; ② under the tensile and compressive stress of the upper and lower interlaced points, resin cracks formed along the direction of the interwoven yarns on the reverse: ③ Kevlar reaches the damage threshold and exhibits irregular pull-out fracture damage.

The damage development of the Kevlar surface on the reverse side under QSI load can be divided into three processes, as shown in Figure 8. Under the tensile force, resin cracks were initially formed inside the Kevlar yarns. Then, tension–compression stress was transmitted along the interlacing points of the yarns, resulting in the extension of the damaged area on the back surface. Eventually, when the Kevlar yarn reached the stress or strain threshold, failure occurred. Due to the toughness of Kevlar yarns and the influence of internal resin cracks in the yarn, irregular pull-out damage appeared on the Kevlar surface. These observations are crucial for understanding the damage mechanism of Kevlar surfaces during the QSI processing of specimens.

### 3.4. Development of Indentation Damage

#### 3.4.1. Acoustic Emission (AE) from Initial Load to Final Failure

The AE signals of plain laminates under QSI testing were measured in real time using the Mistras Micro-Ⅱ digital AE system. In accordance with Ref. [36], K-means clustering associated with principal component analysis (PCA) was used in this study; the optimum cluster number was estimated using the Davies–Bouldin index (DBI), with a huge turning point in the decline of DBI values. Furthermore, the analysis of signal sources related to resin cracking, delamination, and fiber damage was considered in Ref. [37]. The analytical process focused exclusively on mathematical analysis. Finally, the amplitude signals were divided into three levels. The matrix cracking was characterized with an amplitude of 45–52 dB, delamination was 53–63 dB, and fiber pull-out and fiber breakage exhibited amplitudes in the range of 64–100 dB. 

**Figure 9:**
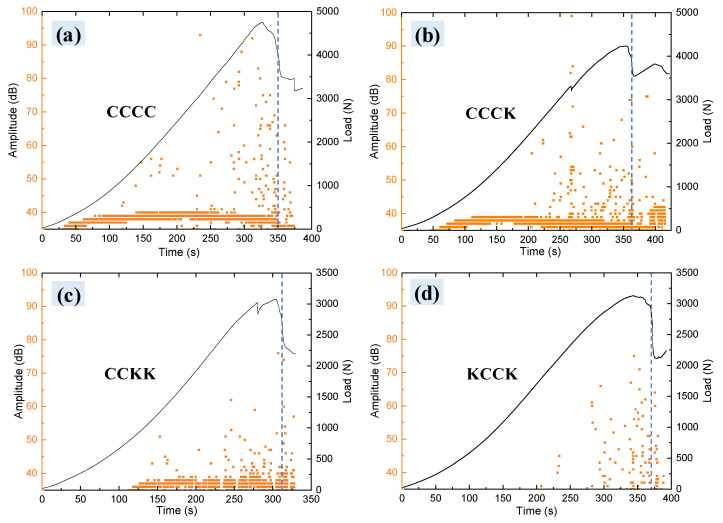

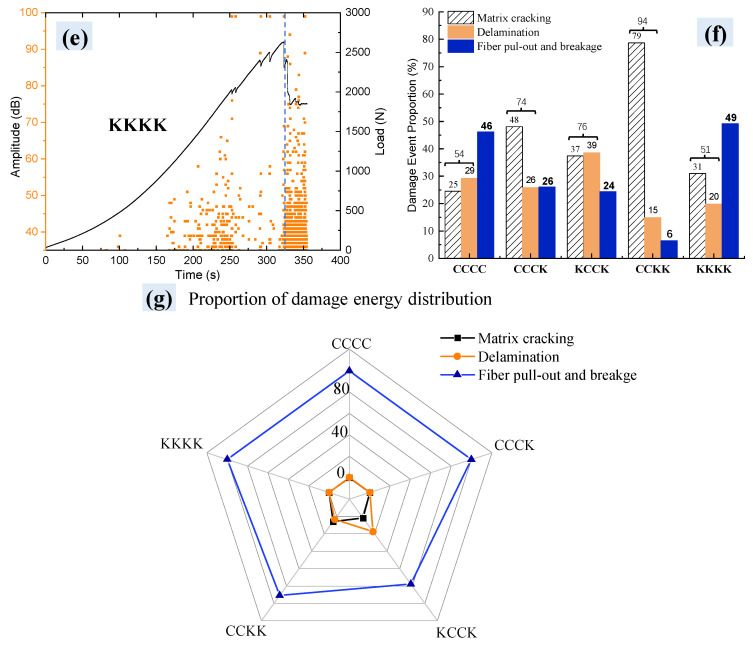
AE response characteristics of the laminates during QSI process: (**a**–**e**) AE amplitude and load–time curves; (**f**) damage event proportional distribution; (**g**) damage energy proportional distribution.

Figure 9 shows the AE response characteristics of the specimens during the QSI process, indicating that matrix cracking occurred first, followed by delamination and finally, fiber failure (fiber pull-out and fiber fracture). However, the final failure time of the carbon/Kevlar hybrid laminates was different with different hybrid plies and intercalation orders. CCCK (Figure 9b, 232s) and KCCK (Figure 9d, 370s) showed longer bearing duration compared with CCCC (Figure 9a, 350s), CCKK (Figure 9c, 312s), and KKKK (Figure 9e, 325s). A longer bearing duration [9] usually means that CCCK and KCCK have a better potential to inhibit damage development. The damage development analysis of the Kevlar on the non-loading side in Section 3.3 indicated that, in comparison to carbon surfaces, Kevlar surfaces tended to develop a large number of diffuse micro-resin cracks; these micro-resin cracks contributed to the dissipation of energy and the distribution of stress, thereby enhancing the overall durability and damage resistance of the material and mitigating the occurrence of structural failure caused by the tensile deformation threshold. Therefore, the single ply of Kevlar on the non-loaded side (CCCK and KCCK) exhibited larger failure deflections, indicating longer load-bearing durations. Conversely, the strength of hybrid structures with multiple plies (two plies or more) of Kevlar on the reverse side was reduced significantly and the tensile fracture stress threshold advanced, leading to earlier structural failure in the CCKK and KKKK structures.

In accordance with references [36,38], the cumulative AE event distribution percentage of laminates under three damage modes in the QSI process was calculated; this is shown in Figure 9f. It was found that KKKK and CCCC underwent mainly fiber damage events such as bundle pull-out and fracture, accounting for 46% and 49%, respectively. The dominant damage event for CCCK and CCKK was matrix cracking, accounting for 48% and 79%, respectively. In all configurations, KCCK showed a damage event mode dominated by delamination and matrix cracking (39% and 37%, respectively). From the perspective of AE damage events, carbon/Kevlar fabric hybrid-reinforced laminates (such as CCCK, KCCK, and CCKK) can effectively reduce fiber damage events involving fiber bundle pull-out and fracture. The difference in results was primarily attributed to the dispersion of resin cracks on the Kevlar surface and the irregular fracturing of the Kevlar fiber bundles. Compared with the carbon surface, there was an increase in the diffuse resin cracks on the Kevlar surface. Due to the dispersed splitting resin cracks within the Kevlar yarn, there was a reduction in the pull-out fractures of the Kevlar fibers. Therefore, the relative proportion of fiber damage events in the Kevlar hybrid structure was reduced.

Furthermore, using Equation (1), the analysis of energy consumption distribution under three damage modes, including cracking, delamination, and fiber damage, in the five structures was conducted; the specific data are presented in Figure 9g:

(1) $$D_{j}=\frac{E_{j}}{E_{T}}=\frac{E_{j}}{\sum_{i}^{n}E_{j}}$$

In the equation, $E_{j}$ represents the energy accumulation under three damage modes, respectively, where *n* = 3, while $E_{T}$ denotes the total accumulated damage energy. Among the five structures, KCCK exhibited the lowest energy consumption associated with fiber damage, followed by the CCKK structure. This indicated that compared with the other structures, the KCCK structure was less prone to fiber damage. The differences in fiber damage are further discussed in the following sections.

**Figure 10:**
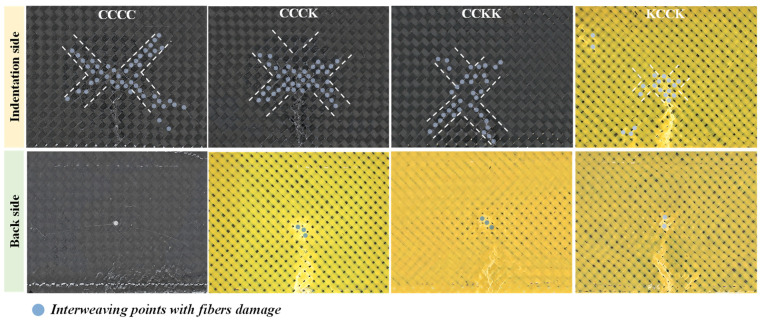
Distribution of fiber bundle damage at interweaving points on the indentation side and reverse side (excluding arch edge damage).

As shown in Figure 10 and Table 4, the fiber bundle damage to the interweaving points of specimens on the indentation and reverse sides was marked and quantified, excluding bulge edge damage. With the increase in the number of Kevlar hybrid plies on the reverse side, the number of interlacing points of fiber bundle damage on the indentation surface decreased, while their number on the non-load side did not significantly change. This indicated that as the Kevlar ratio increased, the amount of fiber bundle damage decreased. As discussed in Section 3.3, brittle fracturing of the entire carbon yarns appeared under stress, due to the brittle characteristics of the carbon fibers, rather than limited fiber pull-out damage to the Kevlar. Therefore, with the ratio of carbon fibers increasing in the hybrid structure, the proportion of fiber damage also increased. Meanwhile, as the number of Kevlar plies increased, the proportion of resin cracks within the Kevlar yarn increased, further leading to a relative decrease in the percentage of fiber damage. Above all, the proportion of energy consumption attributed to the fiber damage of specimens decreased with the increase in Kevlar hybrid plies on the reverse side.

The fiber damage to the KCCK structure was significantly lower than that in the CCKK structure. This phenomenon may be attributed to the fact that the Kevlar yarns on the load side can dissipate energy and effectively disperse stress through forming dispersive resin cracks within the Kevlar yarns, effectively avoiding the formation of linear resin cracks along the main load-bearing yarns. The increased number of linear cracks on carbon fiber surfaces probably resulted in an increased amount of brittle fracturing of the carbon fibers. Thus, the proportion of fiber damage in the KCCK structure was smaller compared with CCKK.

#### 3.4.2. Staged Characterization of Progressive Damage

In general, conventional destructive mechanical experiments can obtain the damage morphology of specimens only after the final failure and it is difficult to observe the evolution process of surface and internal damage simultaneously. Therefore, the QSI process was adopted and divided into four deflection levels to explore damage development, and μCT was applied to obtain the internal damage of specimens during QSI testing at different deflection stages.

(1) Load–deflection curve and macroscopic progressive damage morphology

**Figure 11:**
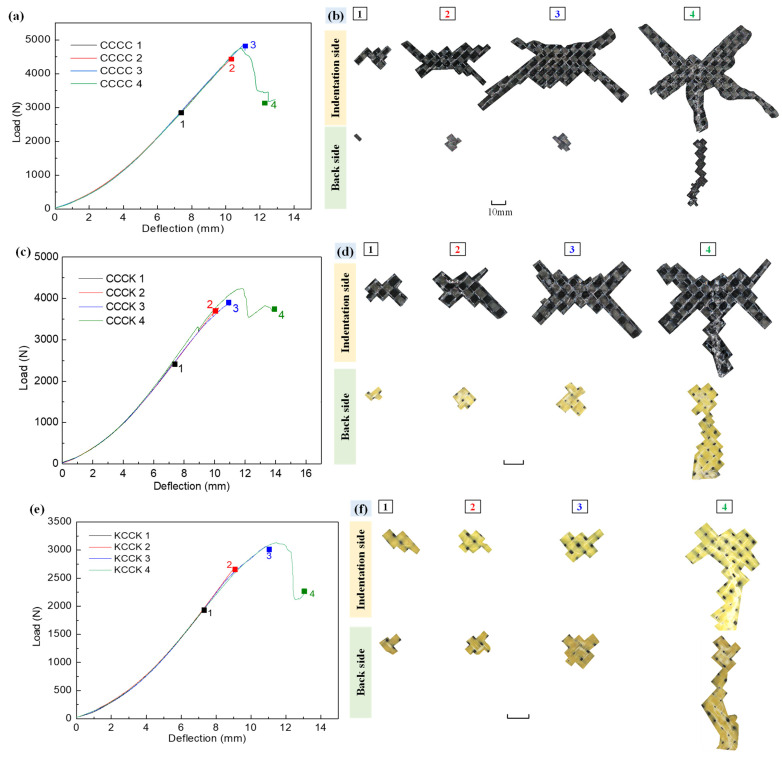

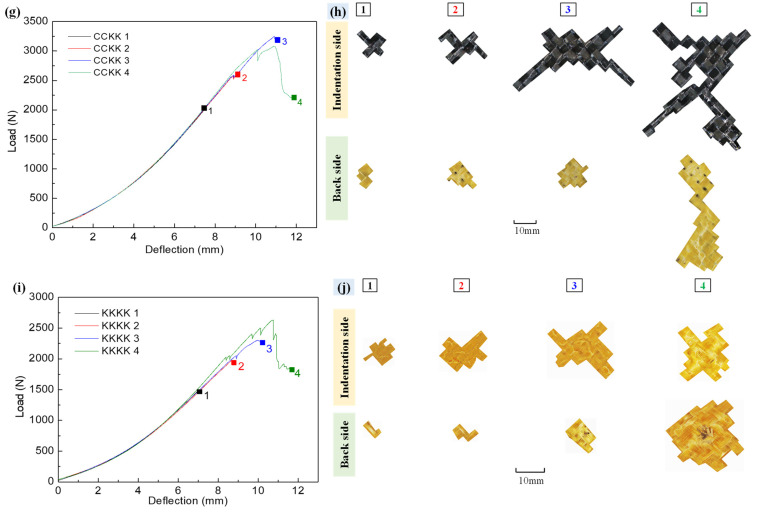
Load–deflection curves and macroscopic damage morphology of the composite specimens at four different deflections from Stage (1) to Stage (4). (**a**,**b**) CCCC; (**c**,**d**) CCCK; (**e**,**f**) KCCK; (**g**,**h**) CCKK; (**i**,**j**) KKKK.

According to the load–deflection curves and AE amplitude characteristics of the specimens, four QSI tests were conducted on the same specimens under different deflections, from which the progressive damage process characterization of the specimens could be obtained (see Figure 11).

Figure 11 demonstrates the load–deflection curves of five different groups of composite laminates (CCCC, CCCK, KCCK, CCKK, and KKKK) under four different deflection stages (1, 2, 3, and 4) and the apparent surface damage areas at the indentation side and reverse side. For the laminates CCCC, CCCK, and CCKK in Figure 11b, Figure 11d, and Figure 11h, respectively, whose indentation surfaces consisted of the carbon fabric ply directly in contact with the indenter, an obvious X-shaped damage area related to the braided load-bearing yarns was observed. However, for the laminates KCCK and KKKK in Figure 11f,j, the Kevlar fabric ply was applied on the indentation surfaces and the X-shaped damage morphology on the top surfaces of the indentation side could be effectively alleviated. Material parameters indicated, as mentioned in Section 2.1, that the Kevlar had a better toughness than the carbon fiber, which could transfer the compressive stress on the indentation surface to a wider range along the interlaced yarns via the dispersive resin cracks, effectively avoiding a large number of resin cracks and fiber brittle fractures caused by the stress concentration of the interlaced yarn on the carbon surface. Therefore, X-shaped damage on the KCCK and KKKK structures was not obvious. Moreover, due to the brittleness of carbon fibers, the carbon fiber yarns on the reverse side found it difficult to propagate stress along the main load-bearing yarn’s direction. This resulted in a significantly smaller damage area on the non-loaded surface with carbon fiber compared to that with Kevlar.

Analyzing the stages of damage morphology of specimens provides a clear understanding of the fluctuations observed in the load–deflection curve. The damage modes of the specimens in four deflection stages are listed in Table 5. In the CCCK and CCKK structures, the damage modes of Stage 2 introduced additional pull-out damage to the Kevlar fiber bundles on the non-loading side compared with Stage 1. This led to a slight decrease in the load in the load–deflection curve of Stage 2. In the KCCK and KKKK structures, Stage 3 showed an increase in the pull-out damage to the Kevlar fiber bundles compared with Stage 2, whereby the load–deflection curve in Stage 3 exhibited more jitter compared with Stage 2, while the load of KCCK in Stage 3 showed a gradual and steady trend compared with Stage 2. Combining the literature review, AE, and analysis of damage morphology, it can be clearly summarized that the jitter in the load–deflection curve was primarily caused by the pull-out damage to the Kevlar fiber bundles on the non-loading side.

**Figure 12:**
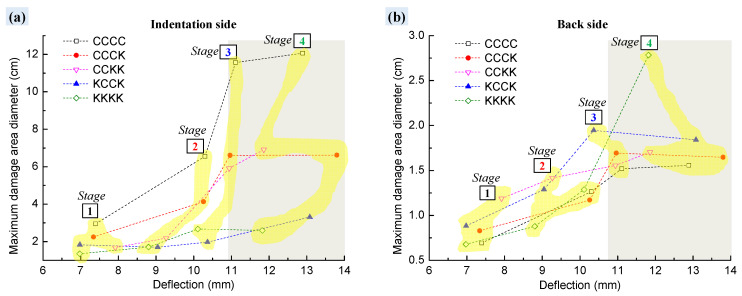
Maximum diameter of the surface macroscopic damage area of composite specimens at four different deflections from Stage (1) to Stage (4). (**a**) Indentation side; (**b**) Back side.

Furthermore, through quantifying the maximum diameter of the damage area on the surface (Figure 12), it was found that the diameter of the damage area on the indentation side (1.3–12 cm in Figure 12a) was larger than that on the reverse side (0.6–2.8 cm in Figure 12b). On the specimen’s indentation surface, direct contact with the indenter initially crushed the resin and, under the control of the four toggle clamps, the pressure propagated along the interlaced yarns to a larger area, causing resin or fiber damage over a larger area. Conversely, under the pressure of the indenter and the four toggle clamps, the reverse surface was fixed to the rectangular support window and damage characteristics similar to those of punching occurred, with the damage area being more concentrated compared with the indentation surface.

In addition, the increase in the number of Kevlar fabric plies was beneficial to reduce the maximum diameter of the damaged area on the indentation side, while significantly increasing the damage area on the reverse side. Compared with the carbon surfaces, Kevlar yarns on the reverse side can distribute the tensile stress over a larger area, resulting in an increase in the damaged area on the back. The pure Kevlar fabric-reinforced laminate KKKK maintained a small diameter of damaged area on the indentation side, as shown in Figure 12a, but, as indicated in Figure 12b, the diameter of the damaged area on the reverse side increased at a significantly fast rate.

(2) Microscopic progressive damage morphology

(1) Porosity of *u*CT

**Figure 13:**
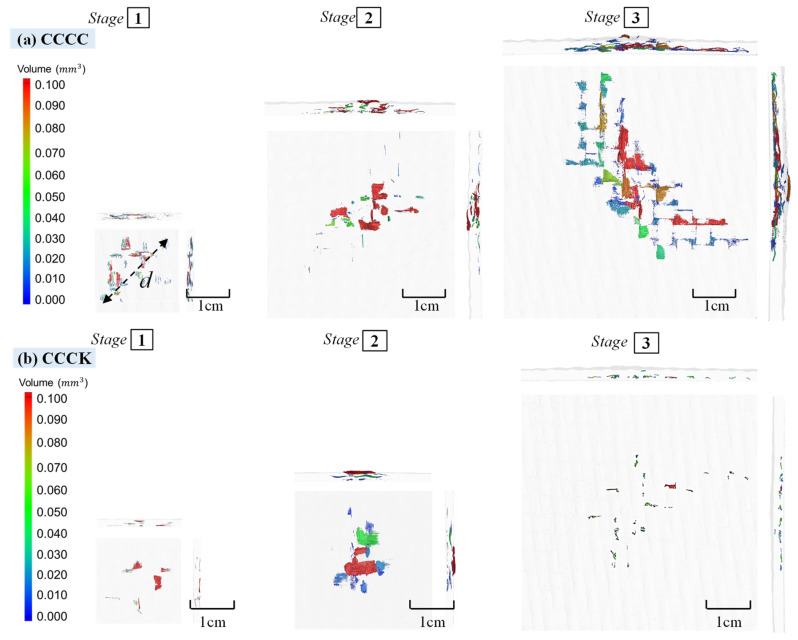

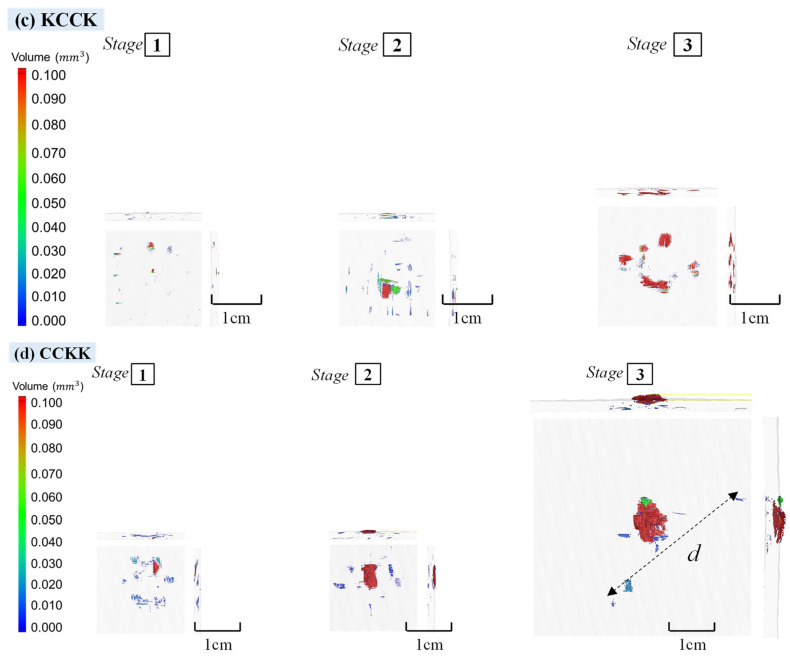
μCT porosity changes of four typical composite laminates (CCCC, CCCK, KCCK, and CCKK) with different carbon/Kevlar hybrid ply and intercalation order under the three different QSI deflections from Stage (1) to Stage (3), before final failure. (**a**) CCCC; (**b**) CCCK; (**c**) KCCK; (**d**) CCKK.

According to the QSI test, the fourth stage included the serious failure of composite laminates with large deflection deformation. In order to understand the process of evolution of the internal damage to the laminates under different QSI deflection states from Stage (1) to Stage (3), before the final failure, the porosity changes of four typical composite configurations, namely CCCC, CCCK, CCKK, and KCCK, were characterized using μCT, as shown in Figure 13.

**Figure 14:**
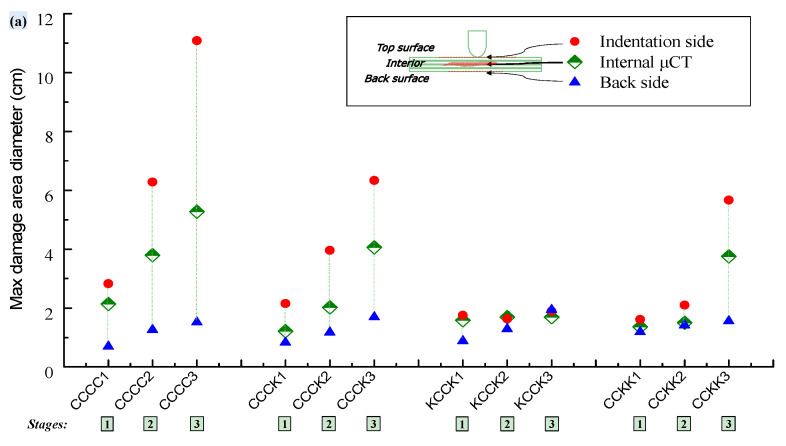

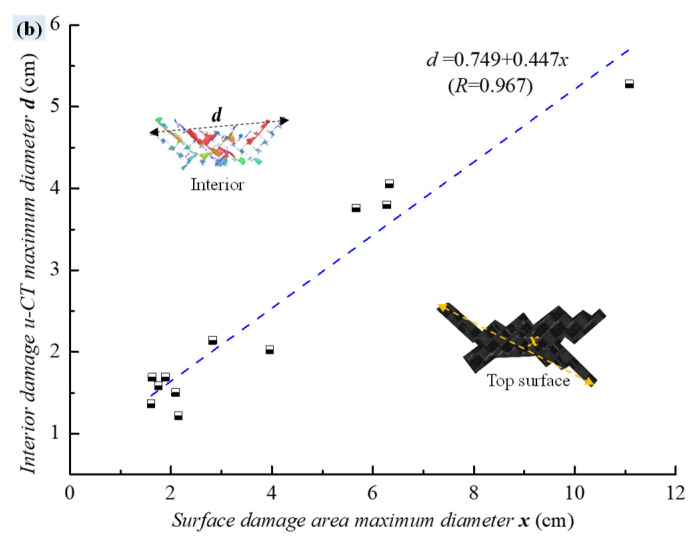
(**a**) The change in the maximum diameter of the internal damage volume observed using μCT and the surface damage area observed from a macroscopic view of CCCC, CCCK, CCKK, and KCCK from Stage (1) to Stage (3); (**b**) the linear correlation between the maximum diameter of the internal damage volume and the damage area on the indentation surface.

The changes in the maximum diameter of the internal damage volume of four typical laminates (CCCC, CCCK, CCKK, and KCCK) from Stage (1) to Stage (3) were analyzed (shown in Figure 14). The results were compared with the maximum diameters of the surface area of damage observed in the macroscopic view, as shown in Figure 11; the quantitative data obtained are shown in Figure 14a.

*d* was defined as the maximum diameter of the internal damage volume observed using μCT. It was found that *d* had a value between the maximum diameter of the indentation side and the reverse side; there was a good correlation between *d* and the maximum damage area diameter *x* on the indentation side (the correlation coefficient was 0.967, as shown in Figure 14b). The linear relationship between *d* and *x* was found to be as follows:

(2) d=0.749+0.447x  (R=0.967)

Therefore, the actual internal damage diameter of the specimen could be inferred from the macroscopic damage diameter on the indentation surface, ultimately determining the actual damage area of the specimen.

Through comparing the results of the KCCK and CCKK structures, it was found that a design with the same hybrid plies but a different hybrid intercalation order could affect the damage development of carbon/Kelvar laminates. In particular, at Stage (3), both the maximum internal damage diameter and the indentation surface of the CCKK with the asymmetric distribution configuration increased sharply. In contrast, KCCK with the sandwich-like ductile–brittle bilaterally symmetric distribution configuration underwent no such phenomenon, further indicating the importance of configuring ductile ply on the indentation side.

(2) Damage development observed using *u*CT

**Figure 15:**
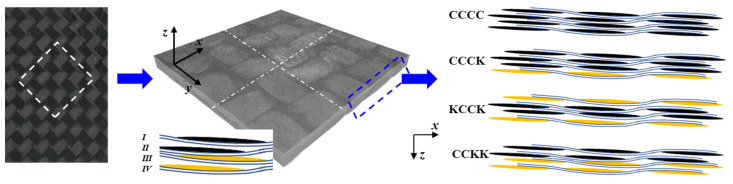
Diagram of the micro-damage observation region and plies with two orthogonal cross sections (x–z and y–z cross sections). Key: definition of fabric layers I, II, III and IV.

**Figure 16:**
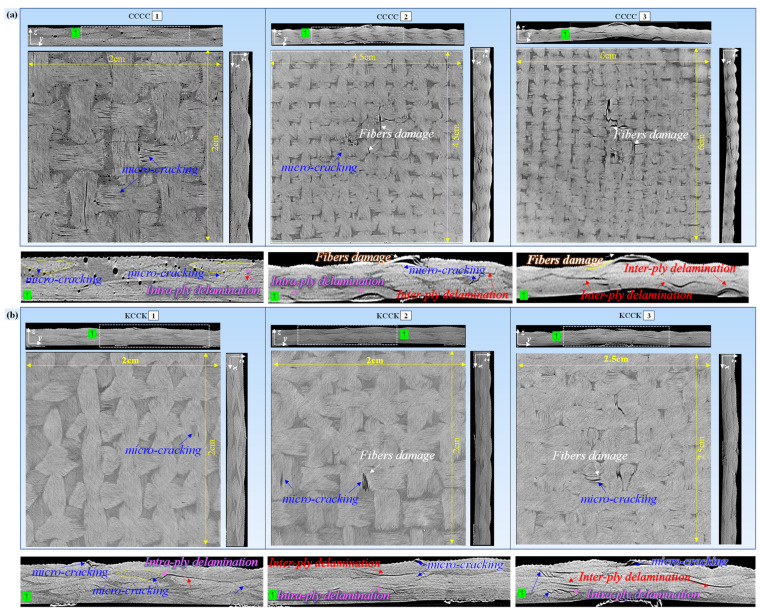

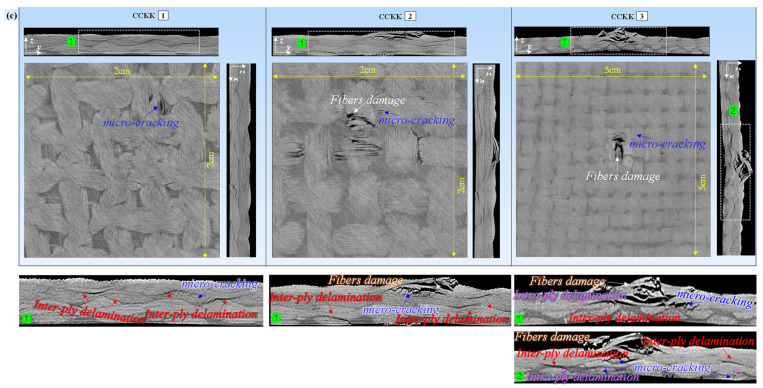
Different section views of internal damage to typical composite laminates (CCCC, KCCK, and CCKK) with different Kevlar hybrid intercalation orders under different QSI deflection from Stage (1) to Stage (3). (**a**) CCCC; (**b**) KCCK; (**c**) CCKK.

As the yarns involved in braiding were closely intertwined with each other, in order to observe the distribution of cracks in different directions within the internally damaged region, two orthogonal cross sections (x–z and y–z cross sections) were drawn, as shown in Figure 15. The resulting different sectional views of the internal damage to typical composite laminates (CCCC, KCCK, and CCKK) with different Kevlar hybrid intercalation orders under three deflections from Stage (1) to Stage (3) are exhibited in Figure 16.

It can be seen from Figure 16 that the internal damage continued to expand from Stage (1) to Stage (3). Stage (1) mainly reflected the delamination cracking of the 4- and 3-ply interlaminar fiber bundles or intralaminar cracking between adjacent fiber bundles with different braiding directions and the same ply. Stage (2) and Stage (3) mainly revealed crack propagation in the x and y directions, beginning from Stage (1). The crack propagation in the thickness direction, further evolving into prominent convex damage, corresponded to the local fiber fracture of the 4-ply or buckling debonding at the indentation surface. It is necessary to understand the damage development described above to configure the hybrid intercalation sequence.

According to Figure 16 and Table 6, the development of delamination within the CCCC structure was primarily caused by intralaminar delamination among interlaced yarns of differing orientations within the 3- and 4-plies. Then, the delamination progressively extended between the 3- and 4-plies, culminating in interlaminar delamination. Concurrently, intralaminar delamination owing to buckling debonding at the 1-ply and interlaminar delamination between the 1- and 2-plies appeared with the expansion of the damage area on the indentation side. In the KCCK structure, delamination commenced with intralaminar and interlaminar delamination both within and between the 3- and 4-plies, subsequently propagating to intralaminar and interlaminar delamination involving the 2-, 3-, and 4-plies. The delamination of CCKK initiated with interlaminar delamination among the 2-, 3-, and 4-plies, thereafter advancing in the thickness direction to ultimately manifest as intralaminar delamination within the 1-ply, along with interlaminar delamination between the 1- and 2-plies. It is worth noting that the KCCK structure exhibited a lower propensity for delamination within the 1-ply and between the 1- and 2-plies compared with CCCC and CCKK. The Kevlar ply on the indentation side was able to effectively disperse compressive stresses at the interlaced points via dispersive resin cracks within the yarn, thereby diminishing the likelihood of intralaminar delamination among the Kevlar yarn interweaving points. Consequently, this effectively controlled interlaminar delamination between the 1- and 2-plies.

**Figure 17:**
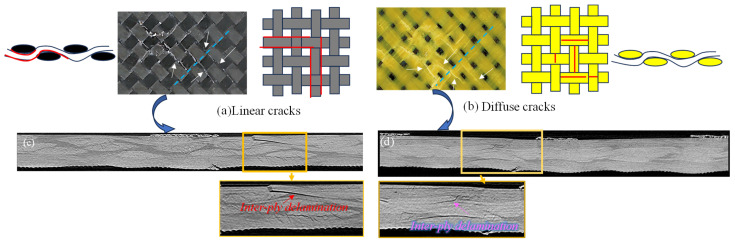
Damage and damage schematic diagram of carbon and Kevlar surfaces on the indentation side of (**a**) CCCK and (**b**) KCCK; (**c**) internal damage to CCCK, Stage 1, and (**d**) KCCK, Stage 2.

As depicted in Figure 17a, significant linear resin cracks along the direction of the main load-bearing yarns formed on the carbon surface at the indentation side. These cracks developed in conjunction with the interlacing and buckling process of the load-bearing yarns, resulting in the formation of delamination cracks within the 1-ply and between the 1- and 2-plies. The cross section of the internal damage to the CCCK structure in its initial stage, as shown in Figure 17c, revealed delamination cracks between the 1- and 2-plies. In contrast, the Kevlar yarns that were arranged on the loading side, as illustrated in Figure 17b, dissipated energy and dispersed stress via forming diffuse resin cracks of internal yarns. This effectively prevented the formation of linear resin cracks and inhibited the development of cracks within the 1-ply and between the 1- and 2-plies. As indicated in Figure 17d, no intra delamination cracks within the 1-ply and no inter delamination cracks between the 1- and 2-plies of the KCCK structure were observed.

Additionally, comparing KCCK and CCKK, which presented the same hybrid ply but different intercalation orders, i.e., symmetrical and asymmetrical, respectively, the KCCK structure exhibited reduced fiber damage on the reverse side. This outcome, consistent with acoustic emission results (there was more fiber damage energy consumption in CCKK than in KCCK), suggested that when the Kevlar fabrics were distributed on the top and reverse plies, presenting a sandwich-like ductile–brittle bilateral symmetrical distribution configuration with external toughness and internal brittleness, both the surface and internal damage propagation of the carbon/Kevlar hybrid composite were effectively suppressed. 

**Figure 18:**
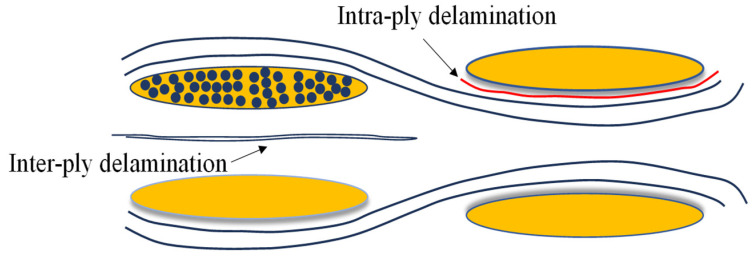
Schematic diagram of the damage development of plain weave under QSI test.

Additionally, combining the internal damage modes revealed using AE and uCT monitoring, the damage during the first stage primarily consisted of resin cracking and delamination. Resin cracking included shear micro-cracks along the yarn direction, intralaminar delamination along the direction of the yarn interlacing settlement, interlaminar delamination, and resin pocket cracks. In the second stage, in addition to resin cracking and delamination, the damage also involved fiber damage, including the compression crushing of fibers on the indentation side or tensile fiber damage on the reverse side. During the third stage, the fiber damage on the reverse side not only continued to develop but also expanded to larger regions, leading to the complete failure of the structure in the fourth stage. The damage development pattern of woven plain laminates under compression can be summarized as follows in Figure 18.

## 4. Conclusions

This work investigated the influence of the carbon/Kevlar fabric hybrid ply and intercalation order on the mechanical index and damage development of composite laminates under QSI load, based on AE and μCT techniques. The specific conclusions are as follows:

(i) As the Kevlar hybrid ply number increased, both plain and triaxial fabric reinforced laminates experienced significant reductions in peak load and stiffness. Specifically, for plain fabric reinforced laminates with 1-ply, 2-ply, and 4-ply Kevlar on the reverse side, the maximum load decreased by 10.72%, 35.42%, and 44.51%, respectively, and stiffness decreased by 7.35%, 25.59%, and 35.55%, respectively. For triaxial fabric reinforced laminates with 1-ply, 2-ply, and 4-ply Kevlar on the back side, the load decreased by 9.97%, 20.69%, and 46.03%, respectively, and stiffness decreased by 6.23%, 19.95%, and 41.43%, respectively. Specimens with the same hybrid ply but different intercalation sequences had a similar stiffness under quasi-static indentation (QSI), with differences of less than 5%.

(ii) Under pressure from the indenter and four toggle clamps, numerous resin cracks and fiber damage occurred along the braided yarns, presenting X-shaped damage on the indentation surface. Kevlar fibers split, leading to irregular Kevlar yarn fractures, whereas carbon yarns exhibited neat fractures with resin crack damage. On the reverse side, Kevlar yarns broke at and near interlaced points along the main load-bearing yarn, while carbon yarns broke only at a single interlaced point.

(iii) Except for the pure Kevlar structure (KKKK), increasing the hybrid ply of Kevlar fabric reduced the percentage of fiber damage events. Resin cracks within Kevlar yarns increased and irregular pull-out damage to Kevlar fibers appeared as the Kevlar hybrid ratio increased, leading to a decrease in the proportion of fiber damage.

(iv) The actual internal damage diameter (d) of the specimen could be inferred from the macroscopic damage diameter (x) on the quasi-static indentation surface as a linear relationship, determining the size of the damaged area. The linear relationship was d=0.749+0.447x (R=0.967).

(v) The KCCK structure showed less delamination between the 1- and 2-plies compared with CCCC and CCKK, due to Kevlar’s effective stress distribution on the indentation side, owing to the dispersed resin cracks within the Kevlar yarns that effectively suppressed intralaminar delamination within the 1-ply and interlaminar delamination between the 1- and 2-plies. Additionally, the KCCK structure exhibited reduced fiber damage on the reverse side, according to uCT observations, consistent with acoustic emission results (there was more fiber damage energy consumption in CCKK than in KCCK). The above statement demonstrates the advantages of the sandwich-like ductile–brittle configuration with bilaterally symmetric distribution in terms of the inhibition of delamination and fiber damage.

## Figures and Tables

**Table 1:** Parameters of bundles.

Type	Tensile Strength (MPa)	Elastic Modulus (GPa)	Elongation (%)
T700	4900	220	1.52
Kelvar49	2814	117	2.14

**Table 2:** The configuration of plain and triaxial fabric-reinforced composite specimens.

Fabric	Configuration		Kevlar Quantity	Ply Ratio (Carbon–Kevlar)	Aerial Density (kg/m^2^)	Thickness (mm)	Fiber Volume Fraction (%)
Plain (±45°)	CCCC	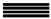	0-ply	4:0	2.41	1.84 ± 0.05	40.7 ± 2.1
	CCCK	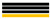	1-ply	3:1	2.32	1.86 ± 0.03	39.8 ± 1.9
	CCKK	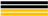	2-ply	2:2	2.30	1.86 ± 0.04	41.7 ± 1.7
	KCCK	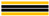	2-ply	2:2	2.30	1.88 ± 0.05	40.8 ± 1.5
	KKKK	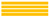	4-ply	0:4	2.28	1.90 ± 0.05	40.4 ± 2.0
Triaxial (±60/0°)	cccc	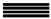	0-ply	4:0	3.47	2.62 ± 0.06	38.2 ± 2.2
	ccck	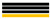	1-ply	3:1	3.41	2.61 ± 0.05	39.5 ± 1.2
	cckk	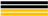	2-ply	2:2	3.22	2.56 ± 0.08	39.1 ± 3.1
	kcck	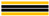	2-ply	2:2	3.22	2.70 ± 0.07	39.7 ± 2.0
	kkkk	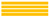	4-ply	0:4	3.22	2.71 ± 0.06	40.3 ± 1.9

**Table 3:** Index changes of carbon/Kevlar hybrid laminates with different Kevlar hybrid plies and intercalation orders (compared with the pure carbon fiber laminate, CCCC or cccc).

Fabric	Index	CCCC	CCCK	KCCK	CCKK	KKKK
Plain	Peak load	0%	−10.72%	−33.86%	−35.42%	−44.51%
	Stiffness	0%	−7.35%	−27.58%	−25.59%	−35.55%
Fabric	Index	cccc	Ccck	kcck	cckk	kkkk
Triaxial	Peak load	0%	−9.97%	−16.31%	−20.69%	−46.03%
	Stiffness	0%	−6.23%	−22.27%	−19.95%	−41.43%

**Table 4:** Numbers of interweaving points of fiber bundle damage.

Type	CCCC	CCCK	CCKK	KCCK
Indentation side	48	41	36	19
Reverse side	1	3	3	2

**Table 5:** The damage modes of plain and triaxial fabric-reinforced composite specimens during the QSI test.

Damage Side	Type	Stage 1	Stage 2	Stage 3	Stage 4
Indentation side	CCCC	○	○√	○√	○ √
	CCCK	○√	○√	○ √	○ √
	KCCK	○ ■＃	○ ■＃	○ ■＃	○ √ ■＃
	CCKK	○√	○√	○√	○ √
	KKKK	○ ■＃	○ √ ■＃	○ √☆ ■＃	○ √☆ ■＃
Reverse side	CCCC	○	○√☆	○√☆	○√☆
	CCCK	○ ■＃	○ √☆ ■＃	○ √☆ ■＃	○ √☆■＃
	KCCK	○ ■＃	○ √■＃	○ √☆ ■＃	○ √☆ ■＃
	CCKK	○ ■＃	○ √☆■＃	○ √☆■＃	○ √☆■＃
	KKKK	○ ■＃	○ √ ■＃	○ √☆■＃	○ √☆■＃

Key: Resin cracks (○); fiber breakage (√); tow splitting (■); fiber tow pull-out (☆); resin cracks along the yarn (＃).

**Table 6:** Delamination distribution of CCCC, KCCK, and CCKK.

Delamination	Stage 1	Stage 2	Stage 3
CCCC	IV and III	IV and III and I	IV and III and II and I
KCCK	IV and III and II	IV and III and II	IV and III and II
CCKK	IV and III and II	IV and III and II and I	IV and III and II and I

## Data Availability

Data are contained within the article.
